# A pictorial key to Southeast Asian species of *Collotheca* (Rotifera, Collothecidae), with a new species from Thailand

**DOI:** 10.3897/zookeys.1294.206505

**Published:** 2026-10-13

**Authors:** Phuripong Meksuwan, Rapeepan Jaturapruek, Thanida Saetang, Yurachat Meksuwan, Wijitra Choedchim, Phannee Sa-ardrit, Diego Fontaneto, Supiyanit Maiphae

**Affiliations:** 1 Science Program (Biology), Faculty of Science and Technology, Phuket Rajabhat University, Phuket 83000, Thailand Science Program (Biology), Faculty of Science and Technology, Phuket Rajabhat University Phuket Thailand; 2 Animal Systematics and Ecology Speciality Research Unit (ASESRU), Department of Zoology, Faculty of Science, Kasetsart University, Bangkok 10900, Thailand Animal Systematics and Ecology Speciality Research Unit (ASESRU), Department of Zoology, Faculty of Science, Kasetsart University Bangkok Thailand; 3 Biodiversity Center Kasetsart University (BDCKU), Kasetsart University, Bangkok 10900, Thailand Biodiversity Center Kasetsart University (BDCKU), Kasetsart University Bangkok Thailand; 4 Department of Fishery Biology, Faculty of Fisheries, Kasetsart University, Bangkok 10900, Thailand Department of Fishery Biology, Faculty of Fisheries, Kasetsart University Bangkok Thailand; 5 Faculty of Technology and Environment, Prince of Songkla University, Phuket Campus, Phuket 83120, Thailand Faculty of Technology and Environment, Prince of Songkla University Phuket Thailand; 6 Faculty of Science and Technology, Princess of Naradhiwas University, Narathiwat 96000, Thailand Faculty of Science and Technology, Princess of Naradhiwas University Narathiwat Thailand; 7 Princess Maha Chakri Sirindhorn Natural History Museum and RSPG Southern Region Network Coordinating Center, Prince of Songkla University, Songkhla 90110, Thailand Princess Maha Chakri Sirindhorn Natural History Museum and RSPG Southern Region Network Coordinating Center, Prince of Songkla University Songkhla Thailand; 8 National Research Council of Italy, Water Research Institute (CNR-IRSA), Largo Tonolli 50, 28922 Verbania Pallanza, Italy National Research Council of Italy, Water Research Institute (CNR-IRSA) Verbania Pallanza Italy

**Keywords:** Collothecids, distribution, infundibulum, sessile rotifers

## Abstract

A new species is described from Thailand, *Collotheca laddae***sp. nov**., and a pictorial key to all *Collotheca* species and subspecies recorded in Southeast Asia is provided. The new species was discovered during a freshwater zooplankton identification workshop. This species is characterized by a five-lobed infundibulum with very small lateral lobes and short vibratile setae. The pictorial key was developed from a review of original descriptions and illustrations of all valid species of *Collotheca* and is designed to facilitate species-level identification based on live specimens. The discovery of *C. laddae***sp. nov**., together with the absence of new regional records for more than a decade, indicates that sessile rotifer diversity in Southeast Asia remains insufficiently explored.

## Introduction

Order Collothecaceae is a taxon of freshwater rotifers in which the typical rotifer corona is lost during metamorphosis and replaced by an expanded, foregut-derived feeding organ known as the infundibulum, which forms a cup-shaped anterior region used in prey capture (Hochberg et al. 2019). The infundibulum, together with the sessile habit of most species, is adapted for ambush predation, in which prey entering the infundibular region are enclosed as the infundibulum folds over them and are then drawn into the lower part of the infundibulum with the aid of the forceps-like apparatus known as the uncinate trophi (Wallace et al. 2015). The order comprises two families. Atrochidae, in which the infundibular margin lacks setae, contains three monotypic genera, *Acyclus*, *Atrochus*, and *Cupelopagis*. Collothecidae, in which the infundibular margin bears setae, comprises two more species-rich genera, *Stephanoceros* (2 spp) and *Collotheca* (48 spp) (Jersabek et al. 2026). The unique features of this taxon should, in principle, make its members readily recognizable and might therefore be expected to have facilitated relatively comprehensive documentation of their taxonomy and biodiversity, particularly in *Collotheca*, the most diverse genus of the order. However, knowledge of collothecid diversity and distribution remains scant in the Oriental region (Segers 2008), including Southeast Asia (Meksuwan et al. 2013), despite the region probably harboring the second-richest freshwater fauna in the world in terms of species richness and endemism (Woodruff 2010). Therefore, further investigation of the taxonomy and diversity of *Collotheca* is warranted to improve our understanding of this distinctive and still insufficiently known group of rotifers.

Among sessile rotifers, comprising the orders Flosculariaceae and Collothecaceae, updated comprehensive species-level keys have recently been produced for several taxa. Regarding Flosculariaceae, Davies et al. (2024, 2025) developed keys to all species and genera of the family Conochilidae (*Conochilopsis* and *Conochilus*) and to most genera of the family Flosculariidae (*Beauchampia*, *Lacinularia*, *Lacinularoides*, *Limnias*, *Octotrocha*, *Pentatrocha*, and *Sinantherina*), while Lafleur et al. (2024) provided a key to *Floscularia*. Together, these studies have substantially improved species-level identification tools for flosculariacean taxa, although further work is still needed for *Ptygura*, the most species-rich genus of Flosculariidae. Regarding Collothecaceae, Davies et al. (2024) also developed keys to genera and species of the family Atrochidae. However, species-level keys for the genera *Stephanoceros* and *Collotheca* in the family Collothecidae remain limited. This gap is particularly important for *Collotheca*, which is the most species-rich genus among sessile rotifers and therefore requires an updated comprehensive key to support accurate species identification and future studies involving genetics, ecology, biogeography and evolution (Fontaneto and De Smet 2015).

Meksuwan et al. (2013) provided dichotomous and formula keys to the species of *Collotheca* recorded in Southeast Asia. In more than a decade since then, only five *Collotheca* species have been reported from three subsequent studies, with no new regional records (Malee et al. 2018; Trinh-Dang et al. 2019; Duong et al. 2020). In addition to the fact that the study of sessile rotifers requires specific procedures (Meksuwan et al. 2011), the shortage of taxonomists is especially critical for rotifers. A lack of local expertise contributes to the “rotiferologist effect”, a phenomenon where recorded rotifer diversity reflects researcher distribution and sampling effort rather than actual species occurrence (Fontaneto et al. 2012). To help address this shortage, we organized a workshop on freshwater zooplankton identification in Thailand (11–15 May 2026), covering copepods, cladocerans, and rotifers. One of the workshop sessions focused on sessile Gnesiotrocha, a group of rotifers that are generally not considered part of the zooplankton, except for a few species and during their juvenile dispersal stage (Davies et al. 2024). During this session of the workshop, we observed several specimens that could be assigned to the genus *Collotheca* but with morphological features that did not match any of the available descriptions of any valid species currently recognized in the genus. The present study provides an updated species-level key to the species of *Collotheca* reported in Southeast Asia, presented as a pictorial key to facilitate the recognition of diagnostic morphological characters. This pictorial key is particularly useful for sessile rotifers, in which species identification often depends on differences in the shape of the infundibulum, the number and form of lobes, and the arrangement of setae. In addition, the study describes a new species of *Collotheca* discovered during the freshwater zooplankton identification workshop in Thailand.

## Materials and methods

### Taxonomic literature review

To confirm that the taxon discovered in this study represents a new species of the genus *Collotheca*, we reviewed the original descriptions and illustrations of all 48 valid species listed in the Rotifer World Catalog (Jersabek et al. 2026). Digital files were obtained through the “Rotifer World Catalog source request” tool and, when available, from open-access online sources. When photographs of living collothecids were available, they were used as the primary basis for representing diagnostic characters. The literature examined included Dobie (1849), Cubitt (1869), Schoch (1869), Collins (1872), Hudson (1883, 1885), Hudson and Gosse (1886), Hood (1888, 1892a, 1892b, 1893, 1894), Anderson (1889), Thorpe (1891), Anderson and Shephard (1892), Rousselet (1893), Wierzejski (1893), Zacharias (1894), Pittock (1895), Milne (1905), Harring (1914), Penard (1914), Monard (1922), Offord (1934), Edmondson (1936, 1940), Varga (1936, 1938), Bērziņš (1951, 1976), Nipkow (1961), Wright (1961), Sládeček (1969), Nogrady (1976), Banik (2002), Meksuwan et al. (2011, 2013), and Franch (2025). Descriptions, identification keys, and illustrations from Koste (1978) were also consulted and compared. These materials were reviewed for taxonomically informative morphological characters used in species identification within *Collotheca*. The characters examined included life habit (sessile or free-swimming); the general shape of the infundibulum; the number, relative size, and shape of lobes; the presence of knobs or other projections; the shape and depth of the ventral sinus; and the length, arrangement, and distribution of setae along the infundibular margin.

### Preparation of the pictorial key

In this study, we defined the Southeast Asian region according to the United Nations Statistics Division M49 geographical classification (UNSD 2026), comprising Brunei Darussalam, Cambodia, Indonesia, Laos, Malaysia, Myanmar, the Philippines, Singapore, Thailand, Timor-Leste, and Vietnam. Original descriptions and illustrations, together with subsequent observations of all *Collotheca* species reported from this region, including the new species described herein, were reviewed and compared. A dichotomous key was then constructed to organize diagnostic characters into a logical sequence of contrasting character states and to guide the preparation of the pictorial key. All line drawings used in the key were redrawn primarily from the original illustrations, supplemented by subsequent records and photographs of living specimens when available. Regarding the drawings used in the pictorial key, *Collotheca algicola* (Hudson, 1886), *C. ambigua* (Hudson, 1883), *C. campanulata* (Dobie, 1849), *C. ferox* (Penard, 1914), *C. stephanochaeta* Edmondson, 1936, *C. tenuilobata* (Anderson, 1889) and *C. trilobata* (Collins, 1872) were drawn directly from photographs, whereas *C. edentata* (Collins, 1872), *C. heptabrachiata* (Schoch, 1869), *C. libera* (Zacharias, 1894), *C. orchidacea* Meksuwan, Pholpunthin & Segers, 2013, *C. ornata* (Ehrenberg, 1830), and *C. pelagica* (Rousselet, 1893) were redrawn and modified from previously published illustrations, namely Koste (1986), Koste (1970), Zacharias (1894), Meksuwan et al. (2013), Koste (1971), and Rousselet (1893), respectively.

### Study site and specimen preparation

**Figure 1.**
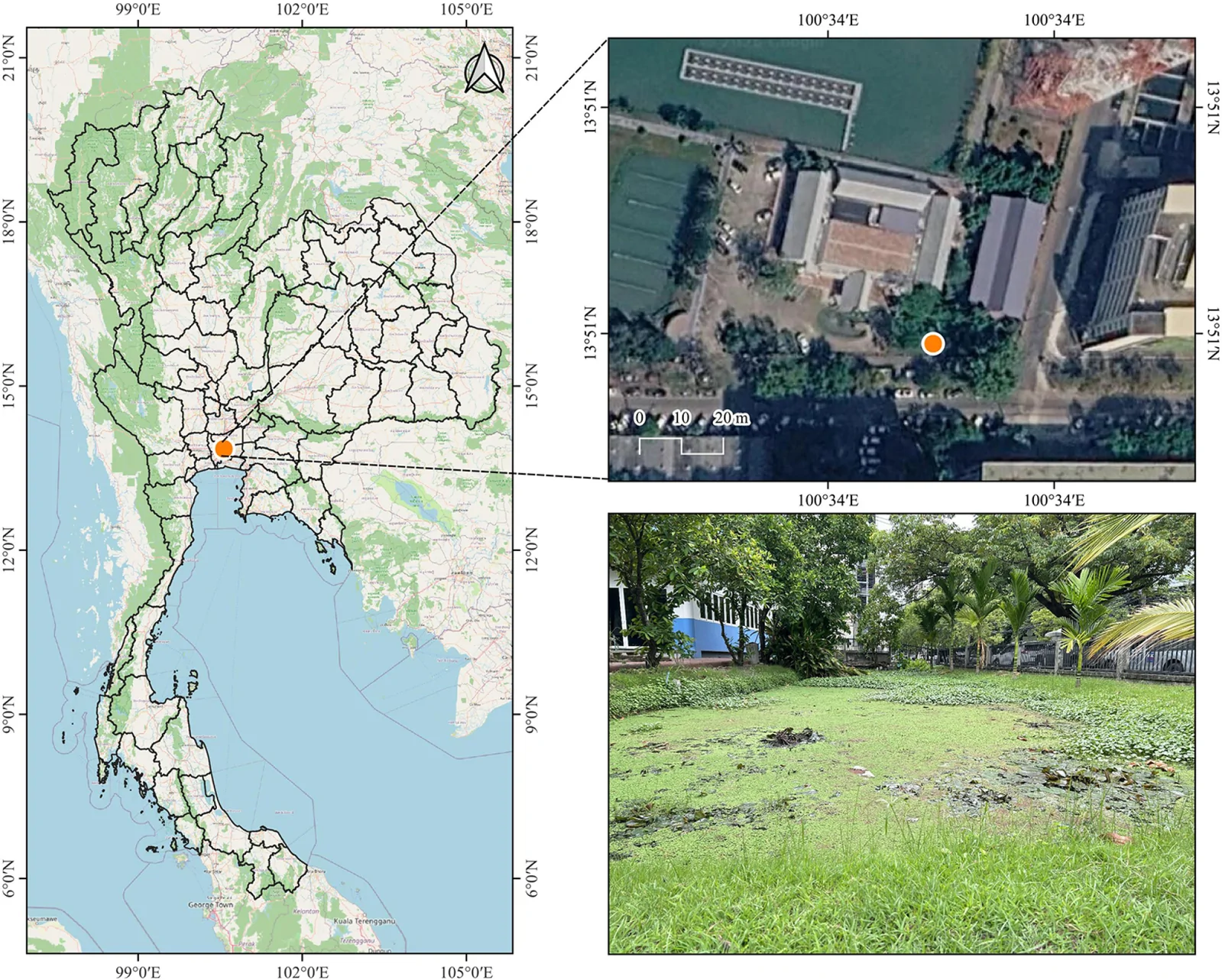
Location and habitat of the study site in Thailand, showing the sampling locality, satellite image, and freshwater pond where specimens were collected.

The study site was an artificial pond at the Faculty of Fisheries, Kasetsart University, Bangkok, Thailand (Fig. 1). Environmental parameters, including water temperature, conductivity, salinity, total dissolved solids (TDS), dissolved oxygen (DO), and chlorophyll *a*, were measured using a YSI EXO1 Multiparameter Sonde, with data recorded via the YSI KorDSS mobile application. Water pH was measured using a YSI EcoSense pH10A Pen, and nitrate concentration was determined using a HORIBA LAQUAtwin NO_3–_ Meter. Physical characteristics of the pond and environmental parameters measured are provided in the type locality section below.

Aquatic plant samples (mainly, *Ceratophyllum demersum* L. and Azolla filiculoides subsp. cristata (Kaulf.) Fraser-Jenk.) used in the rotifer session of the workshop were collected from the study site. The aquatic plants were identified based on e-Flora of Thailand (Forest Herbarium 2019) and the World Checklist of Vascular Plants (Govaerts 2024). Approximately 5-cm-long pieces of aquatic plants were placed in a Petri dish containing water from the sampling site and examined for sessile rotifers under a stereo microscope (Olympus SZ40) equipped with an additional 2X zoom lens. When a sessile rotifer was observed, fine forceps (No. 5) were used to cut the plant fragment on both sides of the attachment site. The plant fragment bearing the attached rotifer was then transferred onto a glass slide containing a drop of water from the sampling site. The preparation was covered with a squared coverslip supported by small pieces of plasticine at the four corners to prevent compression of the specimen. All sessile rotifer specimens encountered during the identification process, including those of the new *Collotheca* species, were identified alive under a compound microscope (Nikon Eclipse E200). Photographs and video recordings were taken using an imaging device (Sony, color video camera) connected to the microscope and a computer. Images were calibrated using a stage micrometer (ERMA, Japan).

Morphological measurements were obtained from expanded live specimens. Measurements included total length (**TL**: foot tip to lobe tip), infundibulum length (**IL**: lobe tip to anterior margin of proventriculus), infundibulum width (**IW**: maximum width), trunk length (**TrL**: anterior margin of proventriculus to foot), trunk width (**TrW**: maximum width), foot length (**FL**: trunk to foot tip), dorsal lobe height (**DH**: base to tip), dorsal lobe width (**DW**: base to base), lateral margin length (**LM**: dorsal-lobe base to ventral-lobe base), ventral lobe height (**VH**: base to tip), ventral lobe width (**VW**: base to base), ventral sinus width (**VsW**: between ventral-lobe bases), ventral sinus depth (**VsD**: ventral-lobe base to sinus base), and seta length (**SL**: base to tip). The lateral lobes of the new species are very small, and their height and width may not be accurately defined; therefore, the lateral lobes were measured as lateral margin length. The number of individuals measured for each character varied according to specimen orientation during imaging, and only specimens positioned at angles that allowed precise measurement of the respective character were included.

Type specimens, together with the plant substrates to which they were attached, were dehydrated through a graded ethanol series (30%, 50%, 70%, 90%, and 95%) to absolute ethanol, cleared in xylene, mounted in Permount mounting medium (Fisher Chemical, Fisher Scientific), and covered with squared glass coverslips supported at the four corners by small pieces of plasticine. Note that all type specimens were preserved with the infundibulum in a contracted condition.

## Results

During the approximately three-hour sessile rotifer identification session of the workshop, we observed an abundant population of *Collotheca ambigua*, a less abundant population of an unknown *Collotheca* species, and a few individuals of *Ptygura mucicola* (Kellicott, 1888) and *P. longicornis* (Davis, 1867) on parts of the plant *Ceratophyllum demersum*. On the roots of Azolla filiculoides subsp. cristata, however, we found *Limnias novemceras* Meksuwan, Jaturapruek & Maiphae, 2018 and *Limnias melicerta* Weisse, 1848, and few individuals of an unknown *Collotheca*. After the unknown *Collotheca* specimens found on both plants were confirmed as not matching any currently recognized species by reviewing the literature for all valid collothecids, we focused on describing the new species and incorporating it into our pictorial species-level key to *Collotheca* species recorded in Southeast Asia. However, molecular data for congeners of *Collotheca* are scarce in the GenBank database, particularly for markers commonly used in species-level delimitation, such as the COI gene, and information on trophi morphology was also unavailable. Therefore, delimitation of the new species was based solely on the external morphology of adult females. The dataset generated from this study has been published through GBIF to provide access to the occurrence and specimen data for further use (Meksuwan et al. 2026).

### Taxonomic account

**Superorder Gnesiotrocha Kutikova, 1970**

**Order Collothecaceae Harring, 1913**

**Family Collothecidae Harring, 1913**

**Genus *Collotheca* Harring, 1913**

#### Collotheca laddae sp. nov.

Taxon classificationAnimaliaCollothecaceaeCollothecidae

B2EF79B2-BD11-5A97-99D5-DC03DBBFFA38

https://zoobank.org/D72C1294-89B4-4A67-9771-92F3891E253E

Figs 2, 3

##### Type material.

**Figure 2.**
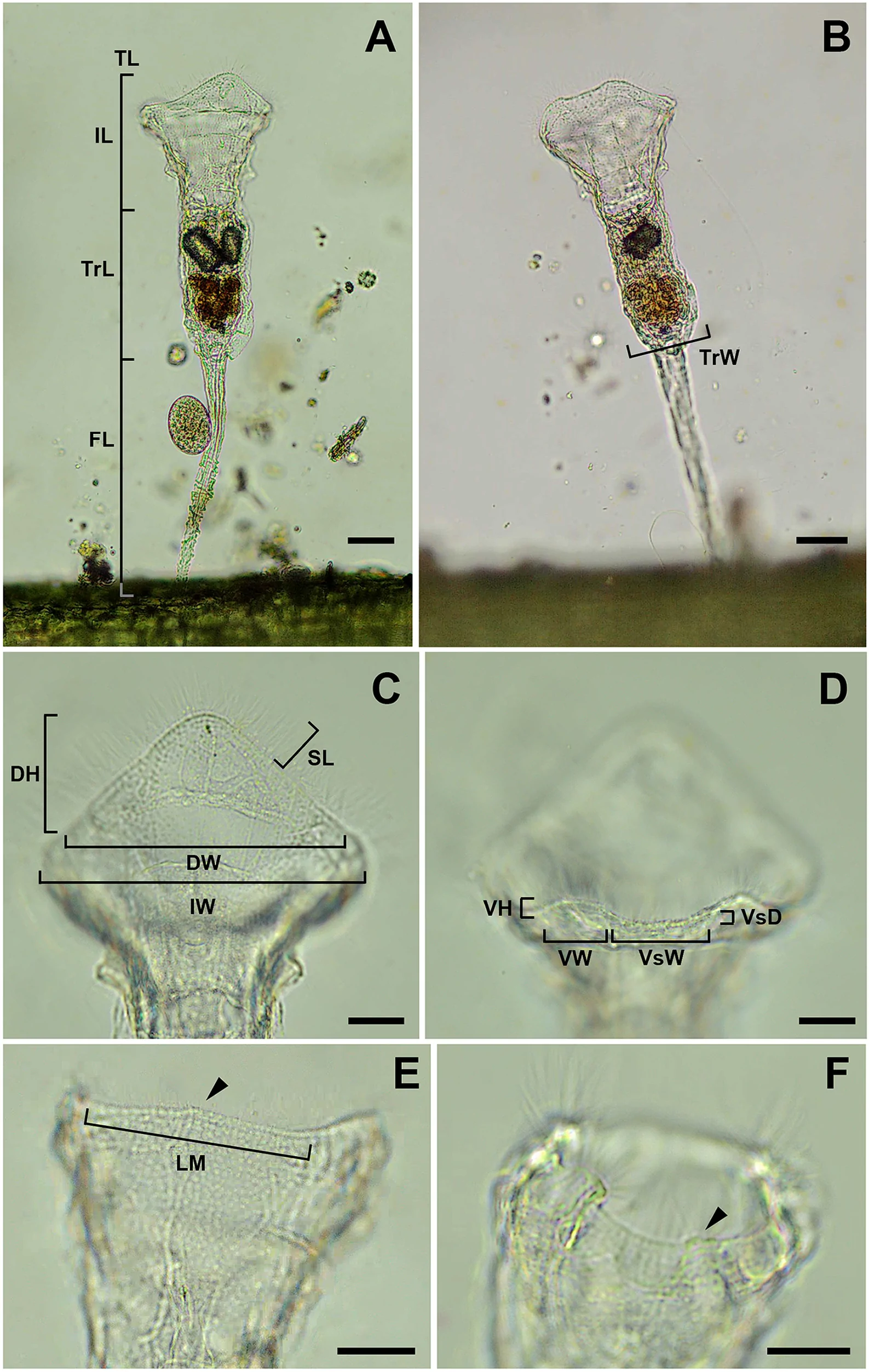
Photographs of *Collotheca laddae* sp. nov. **A**. Habitus, dorsolateral; **B**. Habitus, ventrolateral; **C**. dorsal lobe of infundibulum; **D**. ventral lobes; **E**. lateral lobe of fully expanded infundibulum; **F**. lateral lobe of not fully expanded infundibulum. Abbreviations: DH – dorsal lobe height, DW – dorsal lobe width, FL – foot length, IL – infundibulum length, IW – infundibulum width, LM – lateral margin length, SL – seta length, TL – total length, TrL – trunk length, TrW – trunk width, VH – ventral lobe height, VW – ventral lobe width, VsD – ventral sinus depth, and VsW – ventral sinus width. Black arrowheads indicate the tips of the lateral lobes. Scale bars: 100 µm (**A, B**); 50 µm (**C–F**).

***Holotype*** female (PSUZC-PK5009-01) and three ***paratype*** females (PSUZC-PK5009-02, PSUZC-PK5009-03, PSUZC-PK5009-04), mounted in Permount on permanent microscope slides, are deposited in the Princess Maha Chakri Sirindhorn Natural History Museum, Prince of Songkla University, Songkhla, Thailand. The holotype and one of the paratypes are shown alive in Fig. 2A, B, respectively; however, both the holotype and paratypes are contracted in the permanent slide preparations.

##### Other material.

Six additional females were examined for the description and measurement of characters of the new species.

##### Type locality.

An artificial pond, approximately 21 × 5 × 1 m (length × width × depth), at the Faculty of Fisheries, Kasetsart University, Bangkok, Thailand. WGS84 coordinates: 13.854023°N, 100.569744°E. Specimens were observed on the dominant aquatic plants *Ceratophyllum demersum* and Azolla filiculoides subsp. cristata. At the time of sample collection on 10 May 2026, water temperature was: 31.9 °C, conductivity: 821 µS/cm, salinity: 0.37 ppt, total dissolved solids: 498.97 mg/L, dissolved oxygen: 3.29 mg/L, chlorophyll *a*: 15.84 µg/L, pH: 7.37, and nitrate: 16 ppm.

##### Diagnosis.

*Collotheca laddae* sp. nov. possesses a five-lobed infundibulum, as also recognized in *C. algicola*, *C. ambigua*, *C. bilfingeri*, *C. campanulata*, *C. ferox*, *C. monastica*, *C. moselii*, *C. orchidacea*, *C. spinata*, and *C. torquilobata*. Among these species, *C. laddae* sp. nov. has very small lateral lobes, which distinguish it from species with prominent, distinctly projecting lateral lobes, including *C. ferox* and *C. orchidacea*. It also lacks spines along the infundibular margin, as present in *C. spinata*. In terms of the shape of the dorsal lobe, *C. laddae* sp. nov. has a broad-based, subtriangular dorsal lobe, differing from the uniquely short, U-shaped dorsal lobe of *C. monastica* and from the strongly inwardly curved dorsal lobes of *C. campanulata* and *C. torquilobata*. Moreover, *C. laddae* sp. nov. has small ventral lobes, each ~ 1/4 the width of the dorsal lobe, and a shallow ventral sinus, distinguishing it from *C. moselii*, which bears large ventral lobes, and from *C. algicola* and *C. ambigua*, which have relatively deeper ventral sinuses. *Collotheca laddae* sp. nov. most closely resembles *C. bilfingeri*. The two species can be distinguished by the absence of a dorsal-lobe protuberance in *C. laddae* sp. nov. and by its short setae, which are evenly distributed along the infundibular margin, whereas *C. bilfingeri* bears a protuberance on the dorsal lobe and has very short setae restricted to the tips of the five lobes. Table 1 compares the morphological characters of *C. laddae* sp. nov. with those of other five-lobed *Collotheca* species whose lateral lobes do not project distinctly from the infundibular margin.

**Table 1.** Comparison of morphological characters of *Collotheca laddae* sp. nov. and other five-lobed *Collotheca* species with lateral lobes that do not project distinctly from the infundibular margin.

**Characters**	***C. laddae* sp. nov**.	***C. algicola***	***C. ambigua***	***C. bilfingeri***	***C. campanulata***	***C. monastica***	***C. moselii***	***C. torquilobata***
Dorsal lobe	Medium, subtriangular	Medium, narrow, somewhat triangular	Medium, broad, somewhat triangular	Medium, narrow, somewhat triangular, with a protuberance	Large, rounded, strongly curved inward	Small, U-shaped	Large, uniform width, slightly curved inward	Very large, strongly curved inward
Lateral lobes	Very small, slightly pointed apex	Small, distinctly elevated	Low, broadly rounded	Very low	Low, broadly rounded	Very low	Low, broadly rounded	Low, broadly rounded
Ventral lobes	Small, somewhat semicircular, ~ 1/4 of dorsal lobe	Medium, somewhat triangular, ~ 1/2 of dorsal lobe	Medium, broadly semicircular, ~ 1/2 of dorsal lobe	Small, somewhat triangular, ~ 1/3 of dorsal lobe	Medium, broadly rounded, ~ 1/2 of dorsal lobe	Large, broadly rounded, ~ 2x of dorsal lobe	Large, uniform width, ~ 3/4 of dorsal lobe	Large, broadly rounded, ~ 3/4 of dorsal lobe
Ventral sinus	Shallow, as wide as ventral lobe	Moderately deep, concave	Deep, broadly U-shaped	Shallow, as wide as ventral lobe	Shallow, as wide as ventral lobe	Deep, broadly V-shaped	No data	Moderately deep and narrow
Setae length and distribution	Short, continuous around the infundibulum margin	Long, continuous around the infundibulum margin	Long, not continuous around the infundibulum margin	Very short, on tips of lobes	Long, continuous around the infundibulum margin	Short, absent from lateral sides of dorsal lobe and ventral sinus	Long, continuous around the infundibulum margin	Long, on tips of lobes
Infundibulum/trunk width	~ 1.5x of trunk width	~ 1.5x of trunk width	~ 1.5x of trunk width	~ 1x of trunk width	~ 2x of trunk width	~ 2.5x of trunk width	No data	~ 1.5x of trunk width
Foot length	~ 1.5x of trunk length	~ 2x of trunk length	~ 1.5x of trunk length	~ 1x of trunk length	> 2x of trunk length	~ 1x of trunk length	~ 1.5x of trunk length	~ 2x of trunk length
Peduncle	Short	Short	Short	Short	Short	Short, conical	Short	Short
Trophi and SEM availability	Two pairs of uncinal teeth; SEM not available	Two pairs of uncinal teeth; SEM not available	Two pairs of uncinal teeth; SEM not available	No data	Two pairs of uncinal teeth; head stout, without a medial groove; SEM available (Meksuwan et al. 2013)	Two pairs of uncinal teeth; head with a medial groove and sharp point; SEM available (Franch 2025)	Two pairs of uncinal teeth; SEM not available	No data

##### Description.

**Figure 3.**
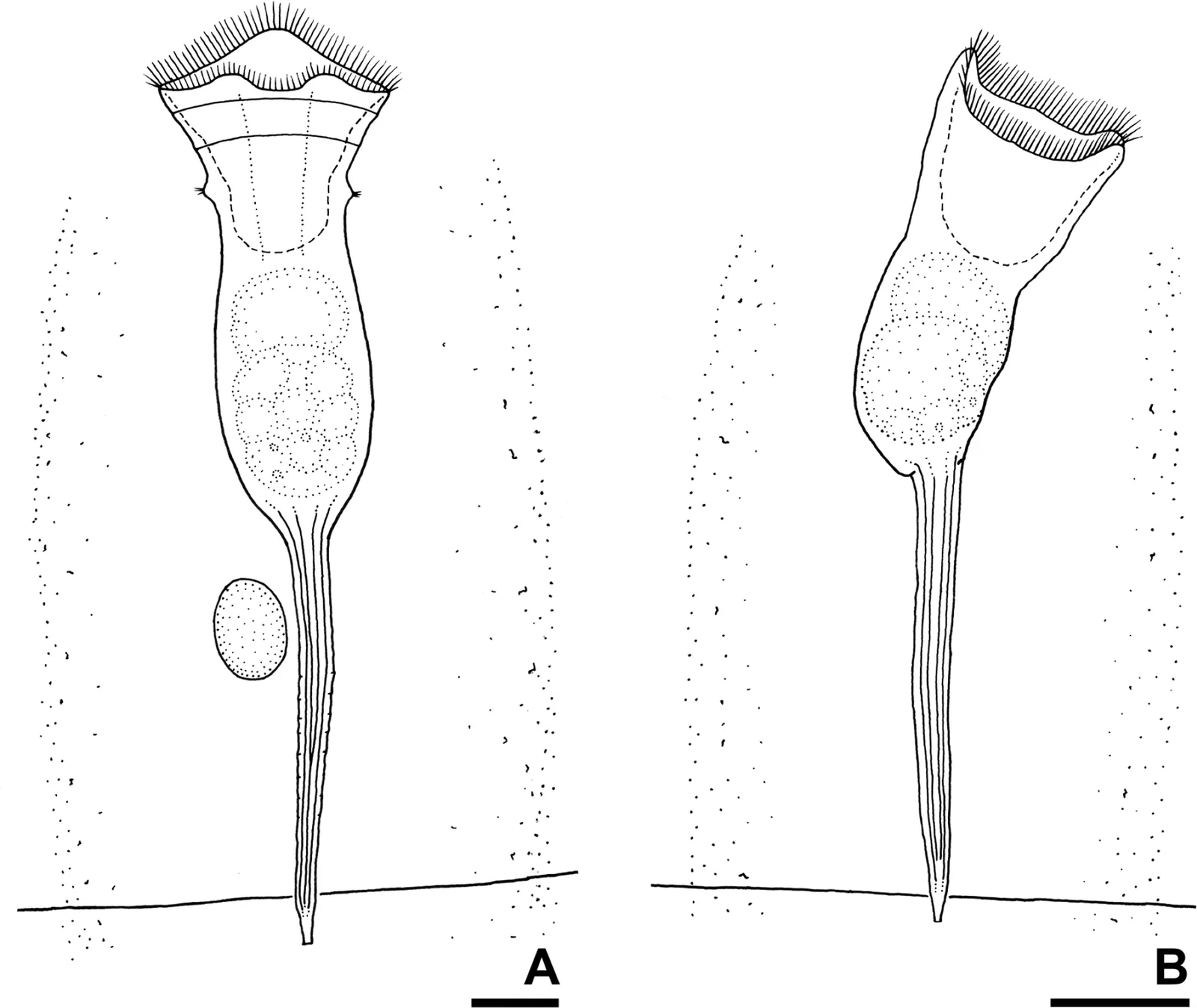
Line drawings of *Collotheca laddae* sp. nov. **A**. Habitus, ventral; **B**. Habitus, lateral. Scale bars: 100 µm.

Most individuals standing upright, with the infundibulum opening directed upward and held from oblique to horizontal (Fig. 2A, B); some individuals slightly curved ventrally. Body elongate; trunk cylindrical to slightly fusiform, widening anteriorly toward the funnel-shaped infundibulum and narrowing posteriorly into the foot. Infundibulum broad, cup- to funnel-shaped, ~ 1.5× as wide as trunk; two transverse folds distinct, one located near the infundibulum opening and the other located posteriorly, parallel to the anterior one (Fig. 3A). Infundibulum five-lobed; dorsal lobe large, subtriangular, with rounded apex (Fig. 2C); two ventral lobes small, somewhat semicircular, each ~ 1/4 of the infundibulum width; ventral sinus shallow, approximately as wide as each ventral lobe (Fig. 2D); two lateral lobes very small, appearing as low, broad-based pointed elevations at the middle of the lateral margins (Figs 2E, 2F, 3B). Setae relatively short, subequal, ~ 1/7 of the corona width, arranged continuously around the corona margin; setae actively vibratile, apparently generating water currents around the corona. Foot as long as the rest of the body, slender, posteriorly tapering, with a short peduncle attached to the substrate. Egg in a clear gelatinous case, located at approximately midlength of the foot (Fig. 2A). Unfortunately, we were unable to successfully prepare the trophi for imaging.

##### Measurements.

Females total length: 290–620 μm (*n* = 8), infundibulum length: 76–220 μm (*n* = 9), infundibulum width: 69–147 μm (*n* = 9), trunk length: 76–155 μm (*n* = 9), trunk width: 56–81 μm (*n* = 9), foot length: 135–303 μm (*n* = 8), dorsal lobe height: 9–73 μm (*n* = 6), dorsal lobe width: 50–121 μm (*n* = 6), lateral margin length: 50–77 μm (*n* = 3), ventral lobe height: 5–10 μm (*n* = 4), ventral lobe width: 18–40 μm (*n* = 4), ventral sinus width: 14–38 μm (*n* = 4), ventral sinus depth: 3–6 μm (*n* = 4), setae length: 13–22 μm (*n* = 8).

##### Etymology.

The specific epithet *laddae* is a feminine noun in the genitive case to honor Professor Ladda Wongrat (current name: Supanin Lohsawadkul), a pioneer of zooplankton studies in Thailand, in recognition of her significant contributions to freshwater zoology and her authorship of important textbooks on zooplankton in Thailand.

##### Distribution.

**Figure 4.**
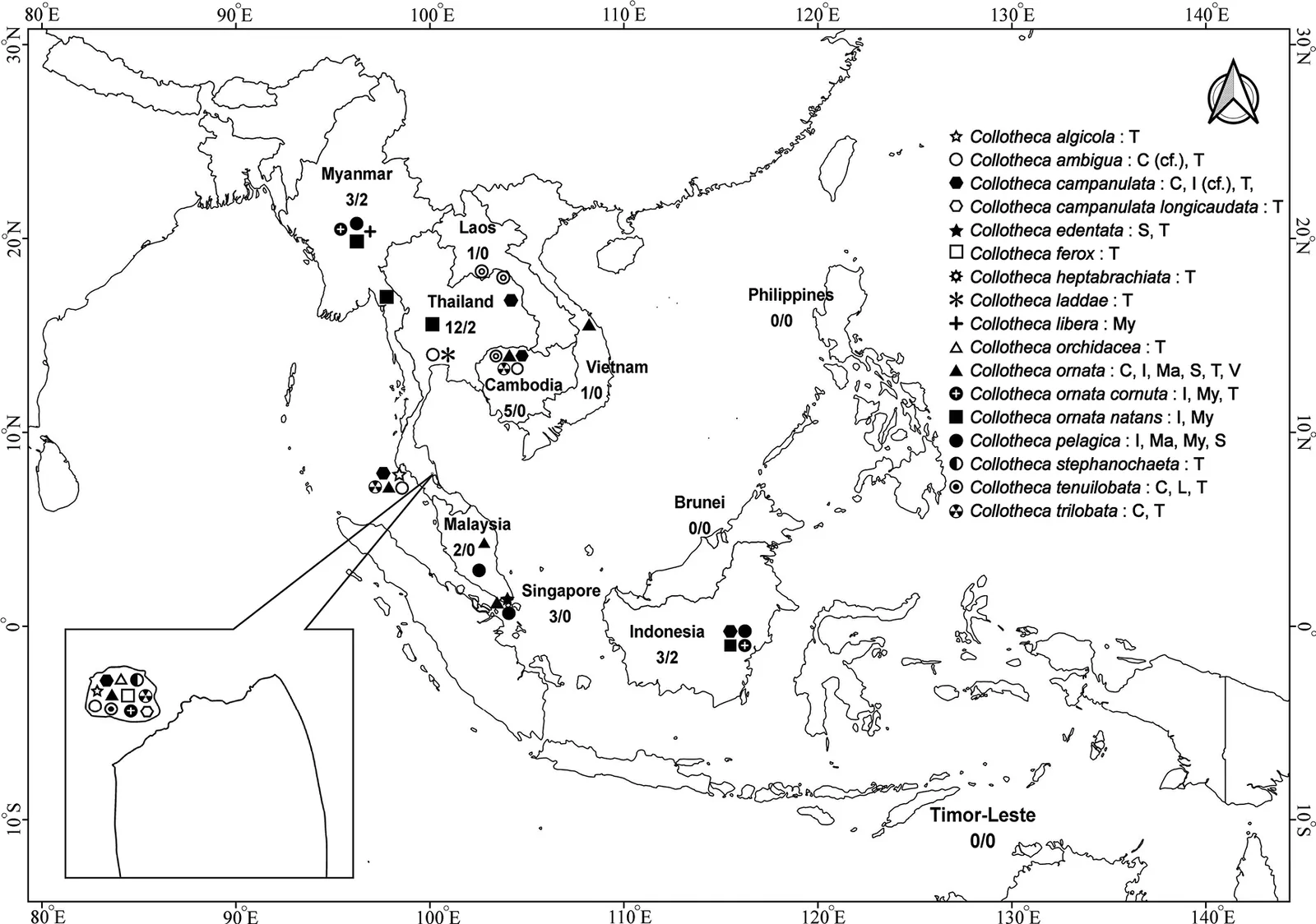
*Collotheca* diversity and distribution in Southeast Asian countries. The first number indicates the total number of species previously reported from each country, whereas the second number indicates the number of subspecies recorded. An asterisk (*) indicated a new species described from Thailand. Distribution of *Collotheca* in Southeast Asian countries indicated: C – Cambodia, I – Indonesia, L – Laos, Ma – Malaysia, My – Myanmar, S – Singapore, T – Thailand, and V – Vietnam. References for *Collotheca* records: Cambodia, Segers et al. (2010); Indonesia, Koste (1988), Sudzuki (1989); Laos, Segers and Sanoamuang (2007); Malaysia, Karunakaran and Johnson (1978), Sudzuki (1989); Myanmar, Koste and Tobias (1990), Aung et al. (2020); Singapore, Karunakaran and Johnson (1978), Sudzuki (1989); Thailand, Koste (1975), Sudzuki (1989), Sanoamuang et al. (1995), Sanoamuang and Savatenalinton (2001), Meksuwan et al. (2011, 2013), Malee et al. (2018); Vietnam, Trinh-Dang et al. (2019), Duong et al. (2020).

The species is currently known only from the type locality. Among Southeast Asian countries, Thailand currently harbors the highest recorded diversity of *Collotheca* species, with 12 species, followed by Cambodia with five species, and Myanmar, Indonesia, and Singapore with three species each. Malaysia has two recorded species, whereas Laos and Vietnam each have one. No *Collotheca* species have yet been recorded from Brunei, the Philippines, or Timor-Leste (Fig. 4).

### Pictorial key to the species and subspecies of *Collotheca* in Southeast Asia

Species of *Collotheca* are freshwater rotifers characterized by animals that are either attached to submerged plants or other substrates, usually within a transparent gelatinous case, or attached within the case while free-swimming in the water column. The anterior end forms a cup- or funnel-shaped infundibulum, the margin of which bears short to very long setae, either continuously or with interruptions. If the observer is uncertain whether the specimen is a rotifer or another invertebrate, attention should be focused on the area near the base of the infundibulum, inside the trunk, where the uncinate trophi may be visible.

**Figure 5.**
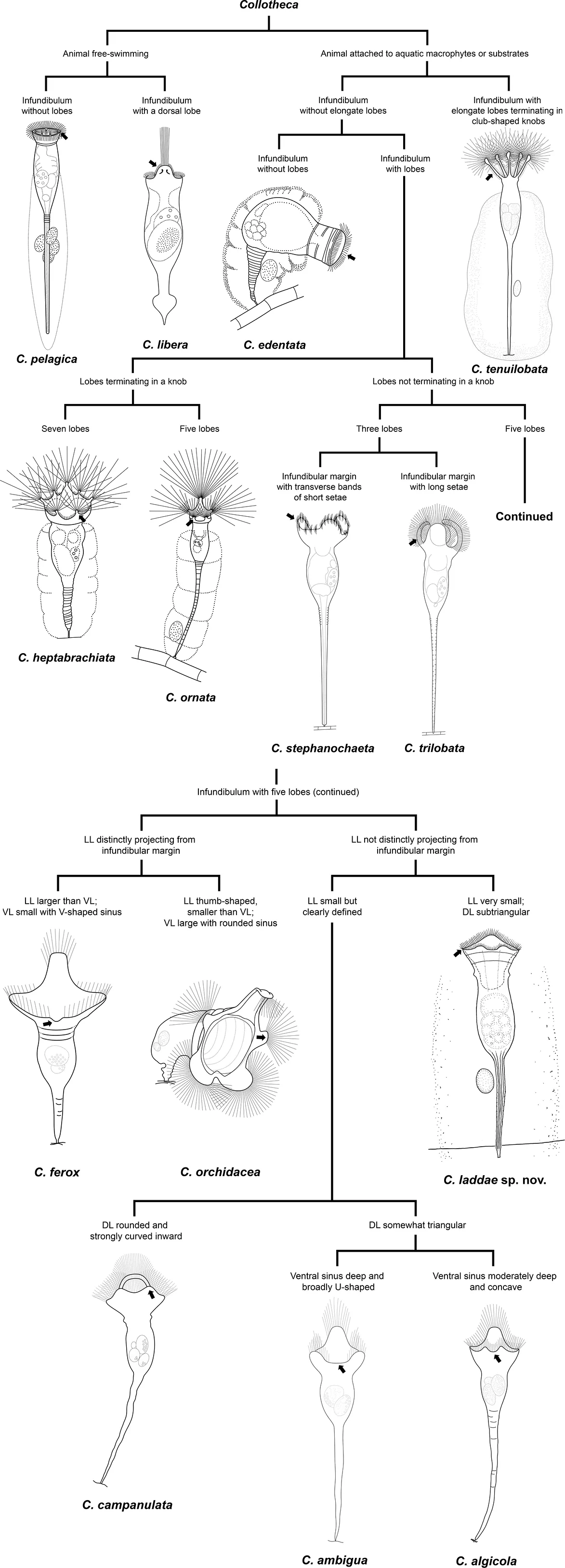
Pictorial key to the species of the genus *Collotheca* in Southeast Asia. Abbreviations: DL – dorsal lobe, LL – lateral lobe, and VL – ventral lobe.

Once the specimens have been confirmed as *Collotheca* rotifers, the first step in the present pictorial key is to determine whether the animals are attached to submerged plants or other substrates or whether they are free-swimming in the water column. Free-swimming specimens are directed to the pelagic *Collotheca* group, whereas attached specimens are directed to the sessile species group. Subsequent steps focus primarily on the number of lobes on the infundibular margin, as well as the shape and presence or absence of knobs on the lobes. The length and distribution of setae are also important diagnostic characters for the identification of *Collotheca* species. These diagnostic characters were integrated into the pictorial key to enable reliable discrimination among Southeast Asian *Collotheca* species. An updated pictorial key to all known Southeast Asian *Collotheca* species is provided to facilitate species identification and future taxonomic studies (Fig. 5).

## Discussion

Studies of sessile rotifers in Southeast Asia are scarce (Meksuwan 2015), and the genus *Collotheca* remains particularly poorly documented. Indeed, no new records of collothecid rotifers have been reported since Meksuwan et al. (2013), a period spanning more than a decade (2013–2026), highlighting a substantial gap in regional research (Fig. 4). Nevertheless, the tools and resources required for regional studies are now more readily available. Meksuwan et al. (2011) provided detailed procedures for the collection and examination of sessile rotifers and emphasized that species-level identification can only be achieved through the study of live specimens. More recently, species-level identification keys for several sessile rotifer taxa have gradually become available through the works of Davies et al. (2024, 2025), Lafleur et al. (2024), and our ongoing efforts to develop keys for the remaining taxa. This is particularly true for collothecid rotifers, for which three complementary identification tools are currently available: (1) the dichotomous key (Meksuwan et al. 2013), (2) the formula key (Meksuwan et al. 2013), and (3) the pictorial key presented in the present study. Moreover, the Rotifer World Catalog (Jersabek et al. 2026) provides the most comprehensive resource currently available for rotifer taxonomy, including valid species names, original illustrations, and a document-request system that enables access to original publications, particularly older and difficult-to-obtain papers. These resources allow researchers to verify taxonomic characters directly from the original descriptions and illustrations. The utility of these resources was illustrated by the discovery of *Collotheca laddae* sp. nov. during a recent workshop mentioned in the introduction. The species was found from material obtained during a single field-sampling event, and its recognition as a new species was achieved after approximately three hours of laboratory observation and examination of live specimens. Together, these tools provide a useful morphological framework for species identification and may facilitate further studies on the diversity and biogeography of sessile rotifers, including collothecids, in Southeast Asia and beyond. Nevertheless, species identification in *Collotheca* remains challenging, and further integration of detailed morphological observations with molecular data will be important for improving species delimitation and identification within the genus.

*Collotheca laddae* sp. nov. possesses relatively short setae. The setae beat actively, generating a strong water current around the infundibulum opening (Suppl. material 1). This current may enhance the transport of algal cells toward the infundibulum, thereby enhancing opportunities for prey capture and engulfment. Similar active beating of the setae has previously been documented in several *Collotheca* species characterized by relatively short setae, both free-swimming and sessile, including *C. mutabilis* (Hudson, 1885), *C. tubiformis* Nipkow, 1961, and *C. hoodii* (Hudson, 1883) (Hudson 1885; Hudson and Gosse 1886; Nipkow 1961). In contrast, most *Collotheca* species possess long, largely immobile setae and are generally regarded as ambush predators that capture prey entering the infundibulum. The occurrence of active setal movements in species bearing relatively short setae therefore raises the possibility that these taxa combine ambush predation with a degree of current-assisted suspension feeding. Although this interpretation requires further investigation, such behavior may represent a feeding strategy that deserves further investigation. Based on analyses of 18S rRNA gene sequences, Collothecacea, represented by eight species from three genera (*Acyclus*, *Collotheca*, and *Stephanoceros*), was recovered as a monophyletic lineage. Furthermore, this lineage formed a sister group to several species of Flosculariaceae, including *Beauchampia crucigera* (Dutrochet, 1812), *Limnias ceratophylli* Schrank, 1803, *L. melicerta*, *Ptygura furcillata* (Kellicott, 1889), and *P. mucicola*, collectively referred to as the BPL group by Meksuwan (2015). Given that suspension feeding is widespread among rotifers and characteristic of many members of the BPL group, the active generation of feeding currents by some short-setae *Collotheca* species may represent a plesiomorphic trait retained from suspension-feeding ancestors. Nevertheless, recent ultrastructural investigations have suggested that the collothecid infundibulum is derived primarily from the foregut rather than from the larval corona, indicating that the setae of the infundibulum are not homologous to the cilia in the corona of other rotifers (Hochberg et al. 2019). This finding complicates interpretations of the evolutionary origin of feeding mechanisms in collothecids and suggests that any similarity between current-assisted feeding/swimming in collothecids and suspension feeding in other rotifers may not necessarily reflect direct homology of the underlying structures. Thus, the functional and evolutionary significance of active setal movements in short-setae species remains uncertain and warrants further investigation through comparative morphological, behavioral, and phylogenetic studies.

## Conclusions

The finding of *Collotheca laddae* sp. nov. during the examination of aquatic plant samples collected for a freshwater zooplankton identification workshop further demonstrates that the diversity and distribution of sessile rotifers in Southeast Asia remain insufficiently explored. Beyond the description of a new species, this study provides an updated species-level identification key to the *Collotheca* taxa reported from Southeast Asia and establishes a morphological baseline for future taxonomic revisions and comparative studies. By compiling and illustrating diagnostic characters of regional species, the present work may also help reduce the “rotiferologist effect”, whereby recorded rotifer diversity and distribution are strongly influenced by the availability and expertise of specialist taxonomists.

## Supplementary Material

XML Treatment for Collotheca laddae

## Additional information

### Artificial Intelligence (AI) use

The authors accept full responsibility for the content of the manuscript, including the disclosure of any use of AI.

Regarding the use of AI in the preparation of this manuscript, the authors declare the following: GPT

Used for: Language, style and writing

The authors used ChatGPT (OpenAI) solely for grammar checking and language improvement of the manuscript. All scientific content, interpretations, and conclusions were developed, verified, and approved by the authors, who take full responsibility for the final version of the manuscript.

### Author ORCIDs

P. Meksuwan https://orcid.org/0000-0001-5478-0709

R. Jaturapruek https://orcid.org/0009-0009-2170-7906

T. Saetang https://orcid.org/0000-0002-7103-7324

Y. Meksuwan https://orcid.org/0009-0003-5932-8895

W. Choedchim https://orcid.org/0009-0004-4272-4899

P. Sa-ardrit https://orcid.org/0009-0002-8838-8734

D. Fontaneto https://orcid.org/0000-0002-5770-0353

S. Maiphae https://orcid.org/0000-0002-7005-6021

### Data availability

All of the data that support the findings of this study are available in the main text or Supplementary Information.

## Supplementary materials

Supplementary material 1 A video shows the live *Collotheca laddae* sp. nov. Data type mov https://binary.pensoft.net/file/1808034This dataset is made available under the Open Database License (http://opendatacommons.org/licenses/odbl/1.0/). The Open Database License (ODbL) is a license agreement intended to allow users to freely share, modify, and use this Dataset while maintaining this same freedom for others, provided that the original source and author(s) are credited. Phuripong Meksuwan, Rapeepan Jaturapruek, Thanida Saetang, Yurachat Meksuwan, Wijitra Choedchim, Phannee Sa-ardrit, Diego Fontaneto, Supiyanit Maiphae
